# Taste Receptors in Upper Airway Innate Immunity

**DOI:** 10.3390/nu11092017

**Published:** 2019-08-28

**Authors:** Ryan M. Carey, Robert J. Lee

**Affiliations:** 1 Department of Otorhinolaryngology, Perelman School of Medicine, University of Pennsylvania, Philadelphia, PA 19104, USA; 2 Department of Otorhinolaryngology and Physiology, Perelman School of Medicine, University of Pennsylvania, Philadelphia, PA 19104, USA

**Keywords:** chronic rhinosinusitis, nitric oxide, innate immunity, cilia, nasal disease, gustation, respiratory infection, antimicrobial peptides, mucociliary clearance

## Abstract

Taste receptors, first identified on the tongue, are best known for their role in guiding our dietary preferences. The expression of taste receptors for umami, sweet, and bitter have been demonstrated in tissues outside of the oral cavity, including in the airway, brain, gastrointestinal tract, and reproductive organs. The extra-oral taste receptor chemosensory pathways and the endogenous taste receptor ligands are generally unknown, but there is increasing data suggesting that taste receptors are involved in regulating some aspects of innate immunity, and may potentially control the composition of the nasal microbiome in healthy individuals or patients with upper respiratory diseases like chronic rhinosinusitis (CRS). For this reason, taste receptors may serve as potential therapeutic targets, providing alternatives to conventional antibiotics. This review focuses on the physiology of sweet (T1R) and bitter (T2R) taste receptors in the airway and their activation by secreted bacterial products. There is particular focus on T2R38 in sinonasal ciliated cells, as well as the sweet and bitter receptors found on specialized sinonasal solitary chemosensory cells. Additionally, this review explores the impact of genetic variations in these receptors on the differential susceptibility of patients to upper airway infections, such as CRS.

## 1. Introduction

**Figure 1:**
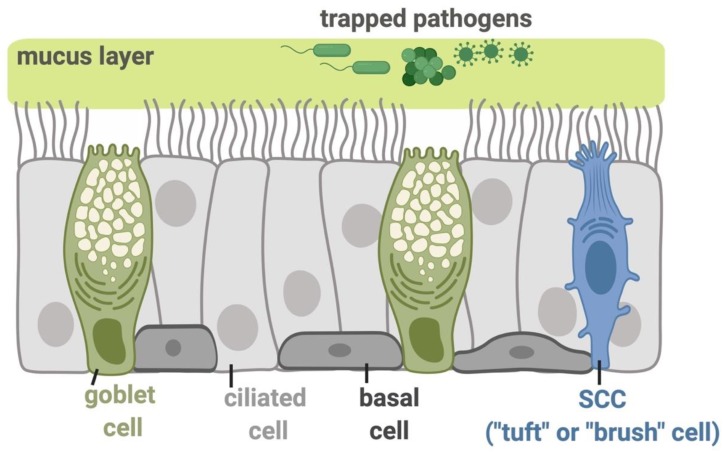
Molecular mechanisms functioning in the airway epithelial innate immunity. The inhaled pathogens, including viruses, bacterial species, and fungi, are trapped in the airway surface liquid (ASL) containing sticky mucus secreted by submucosal exocrine glands and respiratory goblet cells. The trapped pathogens are then eliminated from the airway through mucociliary clearance (MCC), the most important physical defense against these inhaled irritants and microbes. MCC is controlled by the beating of motile cilia and requires the proper regulation of ion and fluid transport by epithelial cells to regulate the mucus viscosity and rheology [5]. The direct killing of pathogens or their inactivation can occur by the secretion of antimicrobial peptides or the production of reactive oxygen species (ROS) and reactive nitrogen species (RNS). The basal cells in the epithelium serve as stem-like cells that can regenerate goblet or ciliated cells during epithelial damage or normal turnover. The ciliated epithelial cells express a gamut of receptors including bitter taste receptors (T2Rs), while solitary chemosensory cells (SCCs; also referred to as tuft or brush cells) express both sweet and bitter taste G protein-coupled receptors (GPCRs), all of which function in regulation of sinonasal innate immunity as described below. All figures were created with BioRender.com.

The sinonasal cavity, which includes the nose and four paired sinuses, serves as the forward line of immune defense against inhaled pathogens [1,2]. The sinonasal epithelial lining utilizes a process termed mucociliary clearance (MCC) as its primary physical defense mechanism to trap and then clear inhaled debris and microbes. This unique mechanism is part of the innate immune system and is dependent on mucus secretion and effective ciliary beating. Additionally, the innate immune system secretes antimicrobial products into the mucus to prevent and neutralize infection [3,4] (Figure 1). When the sinonasal immune defense is impaired, individuals can develop diseases, such as chronic rhinosinusitis (CRS), a multifaceted disease frequently characterized by stasis of secretions from impaired MCC and persistent inflammation and infection in the upper airway [3,5,6]. CRS is one of the most common diseases, affecting more than 16 million Americans annually [7]. It leads to a tremendous economic burden, negatively impacts the quality of life [8,9,10,11,12], and generates almost a quarter of the adult antibiotic prescriptions in the US [13]. Recent research has demonstrated an association between sinonasal innate immunity and sweet and bitter taste receptors, suggesting that taste receptors could be pharmacologically targeted to treat the pathophysiology of CRS and/or other types of respiratory infections.

The sweet and bitter taste receptors, known as T1Rs and T2Rs respectively, are G-protein-coupled receptors that were first discovered on the tongue in type 2 taste cells of the taste bud. T2Rs and T1Rs have been identified in various other organ systems including the upper airway, thyroid, lung, and digestive tract [14,15,16,17,18,19]. While there is canonically only one sweet receptor (made up of a dimer of the T1R2 and T1R3 T1R isoforms), there are 25 T2R bitter receptor isoforms found on the tongue [14,15,16,17,18,19]. The distribution and predominance of T1Rs and T2Rs varies between the different tissues, with some expressing only T2Rs or T1Rs and others expressing both. The role of extra-oral (outside the tongue) taste receptors is currently under intense investigation, but it is now accepted that taste receptors are involved in various local chemosensory functions beyond the originally described neuronal perceptive pathways that originate on the tongue and begin our sensory experience of taste.

Researchers have hypothesized that bitter taste receptors throughout the body may detect bitter compounds secreted by pathogenic fungi or bacteria. The data to initially support this theory was first provided by studies of solitary chemosensory cells (SCCs) in the mouse nose, which express both T1R sweet and T2R bitter receptors [20,21,22,23,24,25,26,27,28,29]. The mouse nasal SCCs exhibited intracellular calcium responses to bacterial acyl-homoserine lactones (AHLs), which are quorum-sensing molecules secreted by gram-negative bacteria, including *Pseudomonas aeruginosa*, a common respiratory pathogen [27,30,31].

Interestingly, there is tremendous genetic variability in the T1Rs and T2Rs, with numerous commonly occurring genetic polymorphisms that underlie the differential taste preferences for foods among different individuals [32,33,34,35,36,37]. Taste receptor genetic variability could also underlie the differences in cellular immune responses, including the clearance of inhaled pathogens in the airway. By doing so, the taste receptor polymorphisms may partially explain the genetic basis of infectious diseases, including respiratory infections [38,39,40]. This review examines the studies primarily in the airway demonstrating that bitter and sweet taste receptors are involved in recognizing various bacterial products. This review also discusses how polymorphisms in one specific bitter T2R receptor correlate with sinonasal infection in CRS, as well as the functional endoscopic sinus surgical outcomes.

## 2. Physiology of Bitter Taste Receptors (T2Rs) in Airway Cilia

In the airway, T2Rs were first identified on the ciliated cells of the bronchial epithelium [41], and later also observed on the sinonasal epithelium [42,43,44], described in more detail below. The activation of bronchial T2Rs by bitter agonists was shown to stimulate a calcium-dependent increase in the frequency of ciliary beating [41]. It was previously thought that motile cilia, with a 9 + 2 microtubule structure and hundreds of cilia per cell, serve only in mechanical roles, such as transporting airway mucus [5]. In contrast, primary cilia, with a 9+0 microtubule structure and usually only one per cell, are involved in the transduction of signals and sensory functions [45]. The expression of functional taste receptors in bronchial motile cilia was an important demonstration that these motile cilia may also function in cellular signaling much like primary cilia.

**Figure 2:**
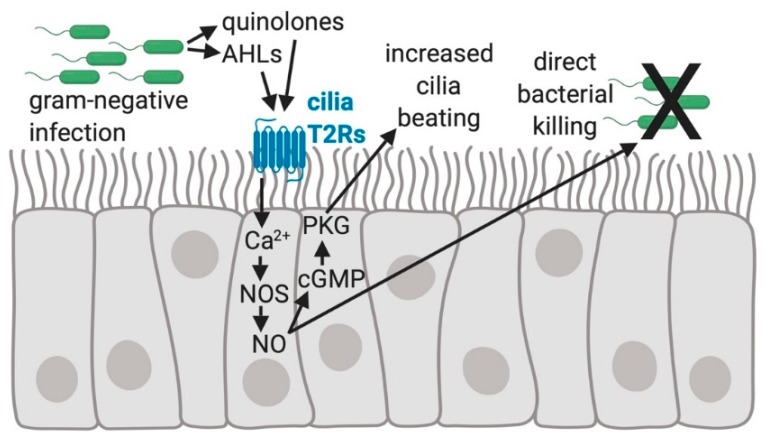
The regulation of human sinonasal epithelial innate immunity by bitter taste receptors (T2Rs). Quinolones and Acyl-homoserine lactones (AHLs) made by gram-negative bacteria, including *Pseudomonas aeruginosa*, activate the T2Rs expressed in human sinonasal cilia, resulting in an elevation of intracellular calcium (Ca^2+^) that stimulates nitric oxide (NO) production by activating nitric oxide synthase (NOS) [44]. NO activates guanylyl cyclase to convert GTP to cyclic-GMP (cGMP), which binds to and increases the activity of protein kinase G (PKG). PKG can phosphorylate ciliary proteins [61,62] causing increased ciliary beat frequency that speeds up mucociliary transport [42,44]. NO diffusion into the airway mucus and surface liquid is bactericidal and possibly damaging to viral or fungal pathogens [44].

Multiple T2R receptors, T2R4, T2R14, T2R16, and T2R38, in the cilia of sinonasal epithelial cells have been identified [44,46,47]. One specific taste receptor isoform, TR38, in airway cilia, is activated by the gram-negative bacterial AHL quorum-sensing molecules initially identified as mouse nasal SCC agonists (Figure 2) [44,48]. In humans, this AHL stimulation of cilia-localized T2R38 leads to robust intracellular nitric oxide (NO) production that occurs through the activation of calcium-dependent nitric oxide (NO) synthase (NOS), likely the endothelial NOS (eNOS) isoform [44]. The intracellular NO activates protein kinase G (PKG) to phosphorylate specific ciliary proteins to increase the frequency of ciliary beating to enhance mucociliary clearance and transport. This calcium and NO signaling involves two canonical components of the classic taste signaling cascade first described in type 2 taste cells, namely the β2 isoform of phospholipase C (PLCβ2) [49,50] and the TRPM5 ion channel [51]. The NO produced also diffuses extracellularly into the airway surface liquid (ASL) where it has antimicrobial properties and kills *P. aeruginosa* [44]. NO produces reactive derivatives, known as reactive nitrogen species (RNS), and both NO and RNS can severely damage bacterial cell walls and membranes, inactivate various bacterial enzymes, and damage bacterial DNA. NO and RNS likely damage fungal pathogen cell walls and viral coat proteins as well [44,47,52,53,54,55,56,57,58]. Therefore, NO/RNS production by the cells of the airway epithelium likely plays an important role in the defense against bacterial, fungal, and viral infection [59,60].

The immune detection of infection by ciliary T2Rs is akin to the more well-studied pattern recognition receptors (PRRs) involved in immunity, such as toll-like receptors (TLRs), which are also found in the airway. PRRs identify molecularly conserved molecular patterns in bacterial, viral, or fungal products, termed pathogen-associated molecular patterns (PAMPs) [1]. The examples of PAMPs include bacterial surface glycoproteins or viral DNA or RNA. The TLR activation results in the up-regulation of mRNA transcription to increase the production of proteins involved in prolonged antipathogen responses. The TLR activation can upregulate the production of antimicrobial peptides including defensins, which occurs over the course of hours [4,63]. Alternatively, the calcium/NO/RNS signaling from T2Rs produced more rapid antibacterial effects, occurring over the course of seconds to minutes [44]. For this reason, these data support the hypothesis that T2Rs represent a fast-acting innate immune response, while these other PRRs, like TLRs, activate more prolonged responses. Thus, these two arms of innate immunity are highly likely to be complementary as they are involved in the different phases of infection.

## 3. Bitter Taste Receptor Genetics and Airway Disease

The magnitude of T2R38 signaling in human sinonasal epithelium depends upon well-described polymorphisms in the *TAS2R38* gene [35,64]. There are two common polymorphisms that are found at high frequency in Caucasian populations. One polymorphism encodes a functional T2R38 receptor and the other a nonfunctional T2R38 receptor as characterized by the ability to detect the T2R38-activating compound, phenylthiocarbamide (PTC). The T2R38 polymorphisms cause different amino acids to be inserted at positions 49, 262, and 296 of the receptor protein. The functional variant of T2R38 contains proline, alanine, and valine (PAV) amino acids and the nonfunctional variant of T2R38 contains alanine, valine, and isoleucine (AVI) at these same amino acid locations, respectively [64]. The loss of the V at the 296 location of the AVI variant likely blocks the activation of the receptor by agonist binding [35,64]. The individuals that are homozygous for the PAV allele (~20% of Caucasian populations) are considered supertasters in that they intensely taste the bitterness of T2R38-specific compounds like PTC and 6-propyl-2-thiouracil (PROP) [64]. The homozygous AVI allele persons (~30% of Caucasians) are non-tasters in that they cannot taste the bitterness of these same T2R38-activating PTC and PROP compounds. Being heterozygous PAV/AVI imparts a variable level of taste that is likely due to the differential expression levels of the transcripts and/or proteins of the PAV and AVI receptor forms [64,65]. Many other haplotypes also exist in non-Caucasian populations, including an AAI polymorphism relatively common in individuals of African ethnicity that also encodes a nonfunctional T2R38 as it also lacks the 296 V residue [66]. T2R38 is, at least, in part responsible for the detection of bitter isothyocyanate compounds in green leafy vegetables, including Brussel sprouts. Therefore, these same *TAS2R38* polymorphisms affect individual taste preferences beyond just preferences for PTC and PROP, raising the intriguing possibility that the genetics of taste are intimately tied to the genetics of infection susceptibility.

The differential respiratory defense responses imparted by these *TAS2R38* polymorphisms was analyzed first by culturing primary epithelial cells isolated from the sinonasal cavities of genotyped PAV homozygous (supertaster), PAV/AVI heterozygous, or AVI homozygous (nontaster) patients. The in vitro level of epithelial cell calcium signaling and NO production correlated with the genetic polymorphisms in *TAS2R38* of each respective patient from which the cells were cultured. Compared with AVI homozygous (nontaster) or PAV/AVI heterozygous cells, the cells from PAV homozygous patients (supertasters) had significantly enhanced calcium and NO signaling in response to both PTC and AHLs that resulted in increased rates of mucociliary clearance and increased bacterial killing [44]. These data indicate that polymorphisms in *TAS2R38* which alter taste perception also alter the responses of sinonasal epithelial cells to gram-negative bacteria. This prompted further investigation of clinical disease phenotypes in CRS patients.

These subsequent clinical studies revealed that PAV homozygous patients had reduced frequency of gram-negative sinonasal infection in vivo compared with AVI homozygous or PAV/AVI heterozygous patients, fitting with the larger T2R38-dependent responses observed in PAV homozygous cells in vitro [44]. The bacteria isolated from AVI homozygous patients have a higher prevalence of forming biofilms in vitro [67] and AVI homozygous patients have a higher incidence of severe CRS disease that leads to medically indicated functional endoscopic sinus surgery (FESS) [68,69]. *TAS2R38* AVI homozygous patients diagnosed with CRS without nasal polyps actually have poorer clinical outcomes after receiving FESS; PAV homozygous patients have better outcomes if they require FESS [70].

In contrast to the above studies, a subsequent analysis of Italian patients did not find a correlation of CRS with *TAS2R38* genotype. However, this study population included individuals with clinical phenotypes of more refractory disease and more enhanced Th2 inflammation than previous studies [71]. A more recent study from Poland found a correlation between PAV or AVI *TAS2R38* and severity of CRS [72]. A Canadian genome-wide association study (GWAS) found that single nucleotide polymorphisms (SNPs) in both *TAS2R38* and *TAS2R13* were more prevalent in CRS versus the control populations [73], while a US study showed that SNPs in both *TAS2R38* and *TAS2R19*, as well as G-beta subunit gene *GNB3*, were correlated with CRS [74]. A study of Australian CRS patients demonstrated that AVI homozygous *TAS2R38* is correlated with the presence of culture-positive bacteria in the noses of these patients [75]. A second Italian study of CRS patients with nasal polyps found a correlation between the lack of T2R38 functionality with gram negative infection in vivo as well as the presence of in vivo biofilms [76]. Intriguingly, preliminary studies suggest that oral taste tests could assist with predicting the susceptibility to certain infections [77].

Other basic science studies have found that T2R38 is also expressed in immune cells [78,79,80,81] and functions in the detection of AHLs in immune cells [78,79]. The bitter taste receptors T2R10 and T2R14, when expressed in HEK293 cells, have been demonstrated to be activated by gram-negative AHLs [82,83]. This review found that airway cilia T2Rs are also potential receptors for two quinolone quorum sensing molecules also produced by *P. aeruginosa* [84,85]. The T2R responses may also be activated when nasal epithelial cells are exposed to conditioned media from *Bacillus cereus* [86]. Together, these mounting clinical and basic science data suggest that T2Rs are bona fide immune recognition receptors that serve a clinically relevant role in innate defense. As such, the differential production of bitter compounds by different species of bacteria, as well as bacteria in different growth stages (e.g., planktonic versus microcolony versus biofilm) may play an important role in controlling the composition of the sinonasal microbiome by allowing epithelial cells to detect invading pathogenic bacteria or to detect when colonizing bacteria have transitioned into potential pathogens [87,88].

Moreover, many natural plant compounds are bitter [89,90], including many compounds in plants used in traditional medicines [91]. Several plant flavonoids activate T2R14 in respiratory cilia and stimulate the protective NO responses described above [47,56,57]. The authors hypothesized that extraoral T2Rs may underlie some of the efficacy observed with some types of homeopathic therapies, much as others have hypothesized that T2Rs may underlie some off-target effects of commonly used drugs [92], which can activate T2Rs expressed in the airway like T2R14 [93]. Understanding if or how compounds in plant-based homeopathic remedies activate extraoral T2Rs may help define the potential mechanisms of action, as well as validate or even optimize efficacy of some of these treatments. If it is found that *TAS2R* gene polymorphisms control efficacy of certain topical infection treatments, these treatments may be better targeted to patients who could derive the most benefit.

## 4. Upper Airway Solitary Chemosensory Cells Express a Different Subset of Bitter Receptors as Well as Sweet Receptors

The T1R sweet and T2R bitter taste receptors are expressed in the upper airway in specialized cells called solitary chemosensory cells (SCCs) [20,21,22,23,24,25,27,28,94,95,96,97], sometimes called tuft cells or brush cells due to an apical microvilli tuft sometimes, but not always, observed [98,99]. SCCs are chemosensory cells with an elongated morphology that are distributed throughout the sinonasal epithelium at a frequency of approximately 1%. The SCC function and signaling appears somewhat similar to the better characterized intestinal tuft cells, named for their apical tuft of microvilli [100,101,102]. The intestinal tuft cells regulate type 2 immunity to parasites [100,101,102]. The tuft cells express chemosensory receptors like T1R3 and succinate receptor SUCNR1 (GPR91) that signal through alpha-gustducin [100,101,102], the G-alpha subunit used by type 2 taste cells [51]. However, much about airway SCC biology, including their signaling and significance to respiratory diseases, still remains to be determined. 

The so-called brush cells with apical tufts of microvilli, very possibly SCCs, were originally identified in the lower airways by electron microscopy in the 1970s in humans [103,104] and even earlier in rats [105,106,107]. The hypotheses of roles for these cells in chemosensation or immune surveillance arose well before any functional data was obtained [98,99]. Recent studies have validated molecular SCC markers like TRPM5 [108] and alpha-gustducin [21], which are also makers of type 2 taste cells [109]. Combined with single cell transcriptomics approaches to characterize airway epithelia and epithelial differentiation at a single cell level [110], researchers are likely to finally begin to unravel the physiology of these cells that were long considered mysterious [98].

**Figure 3:**
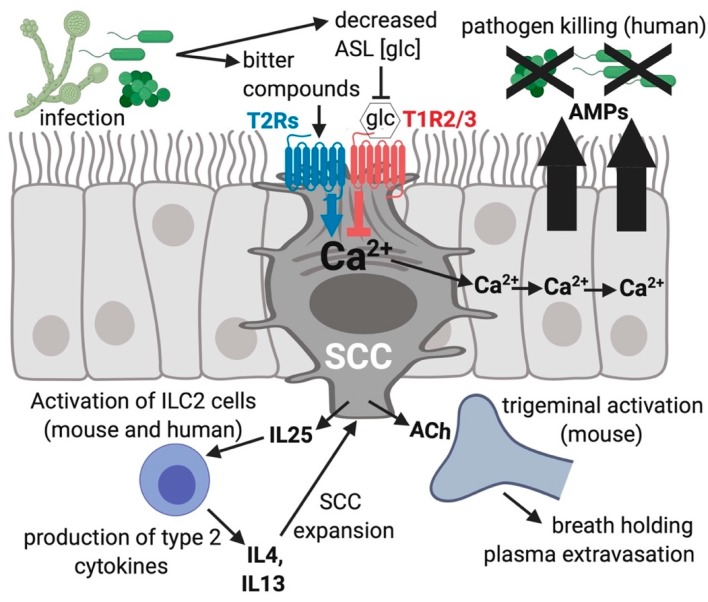
Solitary chemosensory cells (SCCs) function in innate immunity of the airway. Bitter compounds produced during infection can bind to and function as agonists of T2R bitter receptors in SCCs, resulting in a calcium (Ca^2+^) response that travels via gap junctions to connecting ciliated and goblet cells via gap junctions [22]. The calcium propagation causes nearby cells to quickly (≤5 min) secrete stored antimicrobial proteins and peptides (AMPs). One type of AMPs are β-defensins, which directly kill fungi as well as gram-negative and gram-positive bacterial pathogens. During times of more sustained exposure to pathogens, SCCs produce cytokines and chemokines that activate inflammatory pathways which likely recruit specific types of immune cells. Stimulated SCCs can activate trigeminal afferent nerves through acetylcholine (ACh) causing plasma extravasation [23] and reflexive breath holding [25]. Airway surface liquid (ASL) glucose (glc) binds SCC T1R2/3 and reduces the activation of the T2Rs within the same SCC, leading to a decrease in AMP release [22,95]. The different isoforms of T2Rs in SCCs (isoforms 10, 46, 47) and cilia (isoforms 4, 14, 16, 38) respond to different subsets of compounds, potentially allowing the activation of the two different responses at distinct times, which likely depends on which specific infecting organisms and bitter metabolites are present in the airway.

In mice, the activation of nasal SCC T2Rs by bitter compounds can stimulate trigeminal afferent nerves and cause neurogenic inflammation [23] and reflexive breath holding [25]. SCCs are also present in the human nose and sinuses in the inferior and middle turbinates, septum, and uncinate process [20,111]. It was found that in vitro activation of T2Rs in human sinonasal SCCs results in very rapid secretion of antimicrobial peptides, including β-defensins 1 and 2, from surrounding ciliated and goblet cells (Figure 3) [22,95]. Defensins can permeabilize both gram-positive and gram-negative bacteria and kill fungi, like *Candida albicans*, suggesting that the activation of SCC T2Rs would be a broad-spectrum defensive response that could function in immunity against a variety of pathogens. The T2Rs found to be expressed in human SCCs (including T2R10, 46, and 47) are not the same isoforms as those expressed in ciliated cells (T2R4, 14, 16, and 38). Thus, what pathogen products, if any, activate human SCC T2Rs is a question that has not yet been answered. As noted above, mouse nasal SCCs are activated by AHLs [22,95], but human SCCs appear to not be activated by AHLs, which instead activate T2Rs in cilia, as described above.

Interestingly, SCCs co-express T2Rs with T1R2/3 sweet receptors. This is unlike type 2 taste cells, which typically express either bitter or sweet receptors, but not both together within the same cell [109,112]. SCC T1R2/3 is activated by 0.5–1 mM glucose [22,95], a concentration range normally found in the airway surface liquid from tonic leak of serosal glucose across the epithelium [113,114,115,116]. The approximate ASL glucose concentration is 0.5 mM in healthy patients, which is 10-fold below the resting serum glucose concentration [22,95]. This level of glucose is sufficient to reduce T2R calcium signaling within the same SCC and reduce the release of antimicrobial peptides from surrounding cells [22,95]. T1R2/3 in the upper airway can also be activated by pharmacological sweeteners, like sucralose. Notably, T1R2/3 on the tongue is typically activated by higher concentrations (hundreds of mM) of sugar (reviewed in [18,19]). The ability of SCCs T1R2/3 to detect much lower levels of glucose has not yet been explained, and it remains to be determined if one or more additional proteins or secreted molecules play a positive allosteric modulator (PAM)-like role in SCC T1R function.

One hypothesis to explain the existence of the attenuating effects of the physiological ASL glucose levels on this SCC taste receptor system is that it helps prevent false alarms of bacterial infection. The airway T1Rs are calibrated to ASL sugar concentrations present under healthy physiologic conditions. With the onset of infection, the metabolism of glucose by increasing bacterial numbers would be expected to decrease the glucose concentration on the airway surface [113,114,115,116]. If activation of SCC T2Rs by pathogen-produced ligands in the ASL does not occur concomitantly with a glucose decrease, this may reduce the chances of futile killing of the normal nasal flora and commensal bacteria under non-infectious conditions. With a decrease in airway surface liquid the glucose levels signaling the overabundance of bacteria, the T1R2/3 brake on the system is released, allowing for the effective eradication of infection.

The above described mechanism may negatively impact airway diseases in diabetic patients with CRS. Diabetics with elevated serum glucose levels [113,114,115,116] and CRS patients with a decreased barrier function of their epithelium due to chronic inflammation [22,117] both have ≥3–4 fold higher levels of airway surface liquid glucose than normal. The use of sweet receptor antagonists, such as gymnemic acid or lactisole [118], as a topical nasal rinse may restore the full level of T2R immune responses by attenuating the enhanced T1R signaling caused by the elevated glucose in diabetic or CRS patients.

Additional work showed that the sweet receptor (T1R2/3) localized to human nasal SCCs can be activated by at least three bacterially-produced D-amino acids. Some amino acid D stereoisomers, including D-phenylalanine (D-Phe), D-tryptophan (D-Trp), and D-leucine (D-Leu) can activate T1R2/3 on the tongue and thus taste sweet [119]. Cultures of *Staphylococcus aureus* and coagulase-negative *Staphylococcus* were isolated from the noses of CRS patients and found that, when grown in vitro, they produced both D-Phe and D-Leu at concentrations high enough to repress the SCC T2R-mediated responses and the airway epithelial defense by activating T1R2/3 in primary sinonasal cultures in vitro [95]. Other researchers have demonstrated that bacteria produce multiple D-amino acids that could function in cell-to-cell communications or other enzymatic pathways [120]. It has not yet been elucidated if the activation of T1R2/3 by nasal bacteria D-amino acids is a mechanism to allow pathogenic *Staphylococcus* like *S. aureus*, to evade immune detection, or if this allows epithelial cells to recognize but not eradicate commensal *Staphylococcus* species like *S. epidermidis*. D-amino acid production by bacteria and the detection of D-amino acids by host nasal SCCs may be one contributing mechanism to setting the composition of the sinonasal microbiome.

Similar to T2Rs, polymorphisms in the *TAS1R2* and *TAS1R3* genes that make up the T1R2/3 sweet receptor are known to exist, including one in *TAS1R2* resulting in either a valine (V) or isoleucine (I) at amino acid residue 191. The individuals that are V191 homozygous may have increased the incidence of dental caries and hypertriglyceridemia [121,122]. Further studies of polymorphisms in extra-oral taste receptors are needed to better understand how these receptors function in immune surveillance, inflammation, and/or innate defense [109,123] as well as other processes, like beta cell responses to sugars [124,125,126,127,128,129] and neuronal function [130,131,132,133,134]. It has been shown that denatonium-stimulated bacterial killing by sinonasal cells in vitro correlates with CRS patient outcomes [135], supporting a clinical relevance of SCC-induced defensin secretion. The impact of *TAS1R* genetics on airway diseases, including how this alters SCC T2R activation, innate immunity, and CRS outcomes, has yet to be fully elucidated.

SCCs may also be an important reservoir of epithelial IL-25 secretion in CRS [136]. IL-25 is an early cytokine involved in the type 2 inflammatory responses often seen in allergic asthma and CRS with nasal polyps. Further, IL-25 stimulates immune cell production of IL-4 and IL-13 and other type 2 cytokines [88]. SCCs are enriched in the inflamed tissue from patients with allergic fungal rhinosinusitis, and the proliferation and/or differentiation of these cells appears to be stimulated by fungal extract exposure in vitro [137]. A recent study suggested that SCC proliferation may be regulated by leukotriene signaling [138]. While SCCs are predominantly found only in the nose [26] and trachea [97,139] in the mouse airway, they appear more frequently in the distal mouse lung after severe influenza infection based on immunohistological staining for SCC markers [140]. It remains to be elucidated how these cells function in epithelial injury and/or repair during or after fungal or viral infection.

## 5. Conclusions

There is now substantial research to support a role for sweet (T1R) and bitter (T2R) receptors as sensory receptors involved in early innate immune responses in the airway and elsewhere. These taste receptors detect bitter and sweet bacterial metabolites, potentially as a means of distinguishing pathogenic and commensal bacteria. Human infection [141,142] and infectious diseases, such as CRS [38,143,144], have been shown to be impacted by host genetics. Similarly, common taste receptor polymorphisms impact receptor efficacy in detecting agonists, such as secreted pathogen metabolites. Thus, these taste receptors may have a critical, and until-now, overlooked function in contributing to the composition of the airway microbiome.

CRS as a global disease is an important driver of the ongoing emergence of antibiotic resistant bacteria, the so-called superbugs. This enhances the need for alternative pharmacotherapies for managing CRS without the use of conventional antibiotics [13,145,146,147]. By using endogenous T2Rs or other receptors to stimulate endogenous immune defenses, infections may be able to be cleared without resorting to antibiotic therapies. Beyond their role in immunity described in this review, the sweet and bitter receptors regulate the absorption of nutrients in the intestine [148,149,150,151,152,153,154,155,156,157,158], cellular metabolism [159,160], fertility [161], hormone secretion [124,125,126,127,162,163], and likely other physiological processes. Thus, the upper airway is just one organ where extra-oral taste receptors could be targeted to treat disease. Many other diseases may benefit from activating or inhibiting these extraoral bitter or sweet receptors. 

Within the context of the airway, T2Rs also regulate bronchial smooth muscle dilation and may thus be the potential therapeutic targets in asthma [164]. The T1R sweet receptors may be expressed in adipocytes and regulate differentiation [165], though another study suggests artificial sweeteners may have T1R-independent effects on adipocytes [166]. The literature on extraoral taste receptors is still limited. A recent literature review reported that studies focused on ectopic taste receptors and their potential therapeutic applications remain only a small subset of the published taste receptor-related literature [167]. There is a critical need for further research into endogenous activating agonists, signaling pathways, and physiological outputs of these extraoral taste receptors to better understand their role in human disease.
